# Deep Learning-Based Yoga Posture Recognition Using the Y_PN-MSSD Model for Yoga Practitioners

**DOI:** 10.3390/healthcare11040609

**Published:** 2023-02-17

**Authors:** Aman Upadhyay, Niha Kamal Basha, Balasundaram Ananthakrishnan

**Affiliations:** 1 School of Computer Science and Engineering, Vellore Institute of Technology (VIT), Vellore 632014, India; 2 School of Computer Science and Engineering, Center for Cyber Physical Systems, Vellore Institute of Technology (VIT), Chennai 600127, India

**Keywords:** convolutional neural network, deep learning, Mobile-Net SSD, Pose-Net, posture recognition, tensor flow lite

## Abstract

In today’s digital world, and in light of the growing pandemic, many yoga instructors opt to teach online. However, even after learning or being trained by the best sources available, such as videos, blogs, journals, or essays, there is no live tracking available to the user to see if he or she is holding poses appropriately, which can lead to body posture issues and health issues later in life. Existing technology can assist in this regard; however, beginner-level yoga practitioners have no means of knowing whether their position is good or poor without the instructor’s help. As a result, the automatic assessment of yoga postures is proposed for yoga posture recognition, which can alert practitioners by using the Y_PN-MSSD model, in which Pose-Net and Mobile-Net SSD (together named as TFlite Movenet) play a major role. The Pose-Net layer takes care of the feature point detection, while the mobile-net SSD layer performs human detection in each frame. The model is categorized into three stages. Initially, there is the data collection/preparation stage, where the yoga postures are captured from four users as well as an open-source dataset with seven yoga poses. Then, by using these collected data, the model undergoes training where the feature extraction takes place by connecting key points of the human body. Finally, the yoga posture is recognized and the model assists the user through yoga poses by live-tracking them, as well as correcting them on the fly with 99.88% accuracy. Comparatively, this model outperforms the performance of the Pose-Net CNN model. As a result, the model can be used as a starting point for creating a system that will help humans practice yoga with the help of a clever, inexpensive, and impressive virtual yoga trainer.

## 1. Introduction

Information technology and science [1] are progressing at breakneck speed, making human life more convenient than ever. Everyone currently recognizes the significance of it in daily life. The impact of computers and computer-powered technologies on healthcare and allied disciplines, as well as every other domain, is extensively established. Yoga, Zumba, martial arts, and other hobbies, in addition to standard medical procedures, are commonly recognized as strategies to improve one’s health. Yoga is a set of practices related to a person’s physical, mental, and spiritual well-being that originated in ancient India. Artificial intelligence technologies [2], such as Pose-Net and Mobile-Net SSD, as well as human posture detection, can be effective in incorporating tech yoga. Human body posture detection remains a difficult task despite extensive research and development in the fields of artificial intelligence and computer vision. Human posture detection has a wide range of applications, from monitoring health to enhancing the security of the public. Yoga has become a popular kind of exercise in modern society, and there is a desire for training on how to perform proper yoga poses. Since performing incorrect yoga postures can result in injury and tiredness, having a trainer on hand is essential, and since many people do not have the financial means for an instructor, this is where AI offers guidance. The instructor must correct and perform each pose for the individual, and only then can the practitioner perform each pose. However, in today’s situation, more people have adapted to using online tools for their needs and are comfortable with this in their homes. This has led to demand for a tech-driven yoga trainer. As a result, the automatic assessment of yoga postures is proposed for yoga posture recognition, which can alert practitioners. Pose-Net and Mobile-Net SSD (together named as TFlite Movenet) play a major role in using the Y_PN-MSSD model. A Pose-Net layer takes care of the feature point detection, while the Mobile-Net SSD layer performs human detection in each frame.

The working of the model is categorized into three stages:

1. The data collection/preparation stage—where the yoga postures are captured with the consent of four users who are trained yoga professionals, as well as an open-source dataset with seven yoga poses.
2. The feature extraction stage—by using these collected data, the model undergoes training in which the feature extraction takes place by connecting key points of the human body using Pose-Net layer.
3. Posture recognition—using the extracted features, yoga postures are recognized using the Mobile-Net SSD layer, which assists the user through yoga poses by live-tracking them as well as correcting them on the fly. Finally, the performance of the Y_PN-MSSD model is analyzed using a confusion matrix and compared with the existing Pose-Net CNN model.

In the past, many researchers have proposed models for tech yoga practices, and these are discussed below under Section 2. The methodology to recognize yoga postures is given in Section 3. By incorporating the given methodology, the data were prepared, trained, and tested on the proposed model, which is explained in Section 4. Finally, as detailed in Section 5, the results were analyzed and compared with those of the existing model to justify their performance. Deep learning typically needs less ongoing human intervention. Deep learning can analyze images, videos, and unstructured data in ways that machine learning cannot easily do. One of the biggest advantages of using a deep learning approach is its ability to execute feature engineering by itself. In this approach, an algorithm scans the data to identify features that correlate, and then combines them to promote faster learning without being told to do so explicitly. This proposed work uses a unique fusion of Pose-Net and Mobile-Net for better pose estimation when compared with other contemporary works using conventional CNN networks. Additionally, the combination of Pose-Net and Mobile-Net provides better accuracy when compared with other works. These are discussed in detail in the Materials and Methods section and Experimental Results section, respectively.

## 2. Literature Review

Chen et al. [3], in their paper titled “PoseNet: A Convolutional Network for Real-Time 6-DOF Camera Re-localization”, demonstrate a monocular, six-degrees-of-freedom delocalization system that is reliable and works in real-time. Graph optimization is not required, however, as additional engineering would be needed. Their system uses CNN to infer the six poses from a camera shot of a single RGB image (in an end-to-end way). The method can run both indoors and outdoors in real time and computes each frame in large-scale outside scenes. It achieved an accuracy of around 2 m and 6 degrees, and it can achieve an accuracy of up to 0.5 m and 10 degrees. This is accomplished with the use of a productive, 23-layer deep ConvNet, proving that ConvNets may be utilized to address challenging, out-of-image-plane regression issues. This was made possible by leveraging transfer learning from large-scale classification data. They demonstrate how the network can localize using high-level features, and its resistance to challenging lighting, motion blur, and various intrinsic camera points of view.

Islam et al. [4], in their paper “Adversarial PoseNet: A Structure-aware Convolutional Network for Human Pose Estimation”, implemented a joint occlusion method for a human body, which overlapped frequently and led to incorrect pose predictions when used for human pose estimation in monocular images. These conditions may result in pose predictions that are biologically improbable. Human vision, on the other hand, may anticipate postures by taking advantage of the geometric limitations of joint interconnectivity. Alex et al. [5], in their paper on simple and lightweight human pose estimation, demonstrated using benchmark datasets that the majority of existing methods often aim for higher scores by utilizing complicated architecture or computationally expensive models, while neglecting the deployment costs in actual use. They examine the issue of straightforward and lightweight human posture estimation in this study. Chen et al. [6], in their paper on continuous trade-off optimization between fast and accurate deep face detectors, demonstrated that DNNs, i.e., deep neural networks, are more effective at detecting faces than shallow or hand-crafted models, but their intricate designs have more computational overheads and slower inference rates. They researched five simple methods in this context to find the best balance between speed and accuracy in face recognition.

Zhang et al. [7], in their paper “Yoga Pose Classification Using Deep Learning”, proposed a persistent issue in machine vision that has presented numerous difficulties in the past. Many industries, including surveillance cameras, forensics, assisted living, at-home monitoring systems, etc., can benefit from human activity analysis. People typically enjoy exercising at home these days because of our fast-paced lives, but many also experience the need for an instructor to assess their workout form and guide them. Petru et al. [8], in their paper “Human Pose Estimation with Iterative Error Feedback” presented a deep neural network (ConvNets), a type of deep hierarchical extractor, offering outstanding performance on a range of classifications using only feed-forward neural processing. Although feed-forward architectures are capable of learning detailed descriptions of the input feature space, they do not explicitly describe interconnections in the output spaces, which are highly structured for tasks such as segmenting objects or estimating the pose of an articulated human. Here, they offer a framework that incorporates top-down feedback and broadens the expressive potential of hierarchical feature extractors to include both input and output regions.

Yoli et al. [9]. in their paper “Yoga Asana Identification: A Deep Learning approach”, describe how yoga is a beneficial exercise that has its roots in India, can revitalize physical, mental, and spiritual wellbeing, and is applicable across all social domains. However, it is currently difficult to apply artificial intelligence and machine learning approaches to transdisciplinary domains such as yoga. Their work used deep-learning methods, such as CNN and transfer learning, to create a system that can identify a yoga position from an image or frame of a video. Andriluka et al. [10] used an approach for grading yoga poses presented using computerized visuals representing contrastive skeleton features. In order to assign a grade, the primary goal of the yoga pose classification was to evaluate the inputted yoga posture and match it with a reference pose. The research proposed a contrastive skeleton feature representation-based framework for analyzing yoga poses. In order to compare identical encoded pose features, the proposed method first identified skeleton key points of the human body using yoga position images, which act as an input, and their coordinates are encoded into a pose feature, which is used for training along with sample contrastive triplets.

Belagiannis et al.’s [11] work discusses the introduction of deep-learning-based camera pose estimation, and how deep learning models perform transfer learning based on knowledge gained using generic large datasets. As a result, researchers are able to create more specific tasks by fine-turning the model. They first described the issue, the primary metrics for evaluation, and the initial architecture (PoseNet). Then, they detected recent trends that resulted from various theories about the changes needed to enhance the initial solution. In order to promote their analysis, they explicitly suggested a hierarchical classification of deep learning algorithms that focused on addressing the pose estimation problem. They also explained the presumptions that drove the construction of representative architectures in each of the groupings that were identified. They also offered a thorough cross-comparison of more than 20 algorithms, which was comprised of findings (localization error) across different datasets and other noteworthy features (e.g., reported runtime). They evaluated the benefits and drawbacks of several systems in light of accuracy and other factors important for real-world applications.

Buehler et al. [12] in their paper have proposed a spatio-temporal solution that is essential to resolving depth and occlusion uncertainty in 3D pose estimation. Prior approaches have concentrated on either local-to-global structures that contained a pre-set spatio-temporal information or temporal contexts. However, effective suggestions to capture various concurrent and dynamic sequences and to perform successful real-time 3D pose estimation have not been implemented. By modelling local and global spatial information via attention mechanisms, the authors of this study enhanced the learning of kinematic constraints in the human skeleton, including posture, local kinematics linkages, and symmetry.

Chiddarwar et al. [13] concluded that the Pose-Net CNN model was good for yoga posture recognition and developed an android application for the same. The real time data had been captured and processed to detect yoga postures, which guided the practitioners to perform safe yoga by detecting the essential points. Apart from these models, the proposed Y_PN-MSSD model performed well on the captured postures performed by trained yoga practitioners.

Ajay et al. [14] proposed a system that provided consistent feedback to the practitioner, so that they can identify the correct and incorrect poses with relevant feedback. They had used a data set that consisted of five yoga poses (Natarajasana, Trikonasana, Vrikshasana, Virbhadrasana and Utkatasana), which acted as an input to the deep learning model, which utilized a convolutional neural network for yoga posture identification. This model identified mistakes in a pose and suggested solutions on how the posture can be corrected. Additionally, it classified the identified pose with an accuracy of 95%. This system prevents users from being injured as well as increases their knowledge of a particular yoga pose.

Qiao et al. [15] presented a real-time 2D human gesture grading system using monocular images based on OpenPose, which is a library for real-time multi-person keypoint detection. After capturing 2D positions of a person’s joints and skeleton wireframe of the body, the system computed the equation of motion trajectory for every joint. The similarity metric was defined as the distance between motion trajectories of standard and real-time videos. A modifiable scoring formula was used for simulating gesture grading scenarios. The experimental results showed that the system worked efficiently with high real-time performance, low cost of equipment and strong robustness to the interference of noise.

A blockchain-based decentralized federated transfer learning [16] method was proposed for collaborative machinery fault diagnosis. A tailored committee consensus scheme was designed for optimization of the model aggregation process. Here, two decentralized fault diagnosis datasets were implemented for validations. This work was effective in data privacy-preserving collaborative fault diagnosis. This proposed work was suitable for real industry applications. A deep learning-based intelligent data-driven prediction method was incorporated in [17] to resolve sensor malfunctioning problem. Later on, a global feature extraction scheme and adversarial learning were introduced to fully extract information of sensors as well as extract sensor invariant features, respectively. This proposed work was suitable for real industry applications.

A two stream real-time yoga posture recognition system [18] was developed and a best accuracy of 96.31% was achieved. A yoga posture coaching system [19] using six transfer learning models (TL-VGG16-DA, TL-VGG19-DA, TL-MobileNet-DA, TL-MobileNetV2-DA, TL-InceptionV3-DA, and TL-DenseNet201-DA) was exploited for classification of poses, and the optimal model for the yoga coaching system was determined based on evaluation metrics. The TL-MobileNet-DA model was selected as the optimal model as it provided an overall accuracy of 98.43%.

Qian et al. [20] proposed a contactless perspective approach, which is a kind of vision-based contactless human discomfort pose estimation method. Initially, human pose data were captured from a vision-based sensor, and corresponding human skeleton information was extracted. Five thermal discomfort-related human poses were analyzed, and corresponding algorithms were constructed. To verify the effectiveness of the algorithms, 16 subjects were invited for physiological experiments. The pose estimation algorithm proposed extracted the coordinates of key points of the body based on OpenPose, and then used the correlation between the points to represent the features of each pose. The validation results showed that the proposed algorithms could recognize five human poses of thermal discomfort.

## 3. Materials and Methods

Deep learning is an integral and essential learning-based method (ANNs) that represents the working of neurons in the human brain with the help of weights. This helps in representing the connection between neurons. It is an e-architecture [21], this allows crucial information from images to be learned automatically.

**Figure 1:**
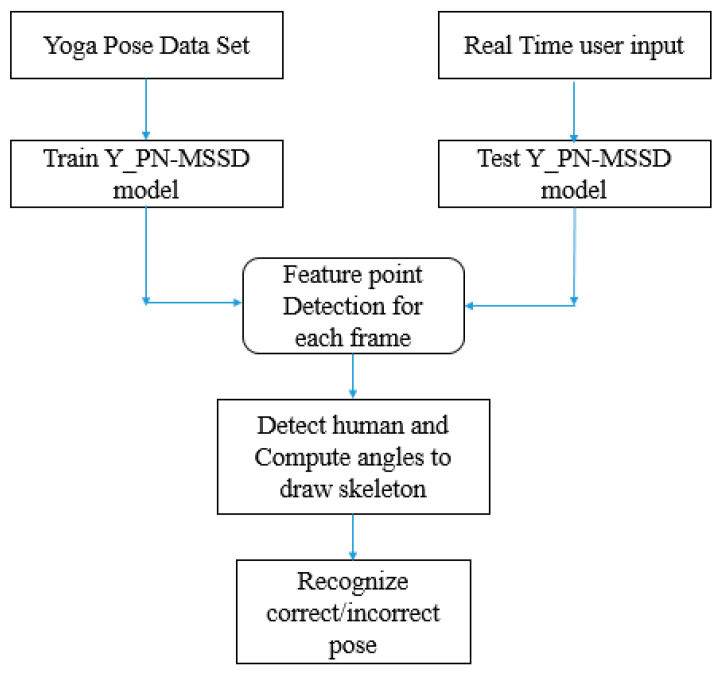
The working of the Y_PN-MSSD model.

**Figure 2:**
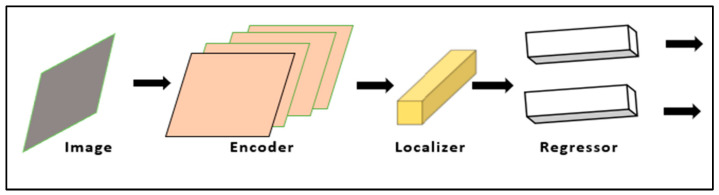
Pose-Net architecture.

**Figure 3:**
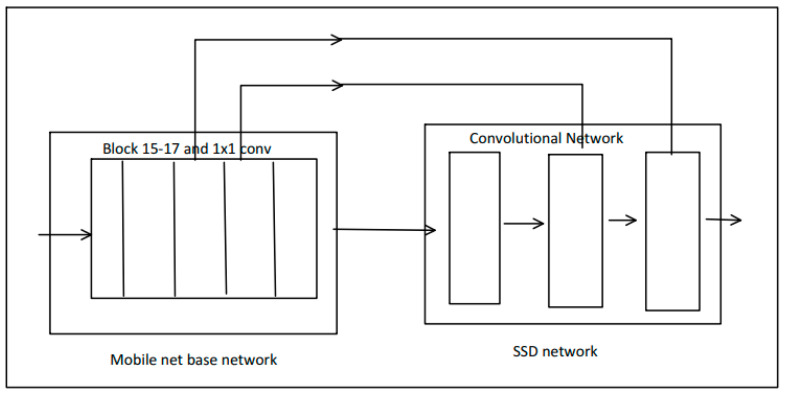
Mobile-Net SSD architecture.

CNN is one of the deep learning models that has been widely utilized for estimating yoga poses. However, they are judged only based on images rather than videos. Here, the proposed Y_PN-MSSD model uses TFlite Movenet for recognizing yoga poses. Initially, an input (yoga pose) has been captured and processed using trained TFlite Movenet, where the Pose-Net detects the feature point from the input and the Mobile-Net SSD detects the person in the captured frames. Finally, the model recognizes the correct/incorrect yoga pose. The working of the model is depicted in Figure 1. The detailed description of Pose-Net and Mobile-Net SSD are given below with their respective architectures depicted in Figure 2 and Figure 3.

### 3.1. Pose Net

It is a deep learning framework that identifies the positions of joints in images or video sequences involving human body movement. The joints are considered as key points, which are indexed by Part ID with a range from 0.0 to 1.0, with 1.0 as the highest score. Among different layers, soft-max activation function, which is at the last layer, has been replaced with a series of fully linked layers. Figure 1 depicts its high-level architecture. The performance of this model [22] depends and differs completely on the device and output stride. Because of this, the model works on posture positions in the entire scale of captured images, regardless of the size. Here, an encoder, localizer and regressor act as three components. The encoder generates the encoding vector, which contains the features of the input image represented as a 1024-dimensional vector. The localizer generates a vector with localized features. Finally, a regressor is used to perform regression on the final position using two linked layers in the model.

### 3.2. Mobile-Net SSD

It is a model that computes the input for making bounding box and categorize them from an input which is also called as object detection model. Single Shot Detector (SSD) in this model uses Mobile-Net [22] to achieve optimized object detection, which will work faster on mobile devices. This model contains offset values (cx, cy, w, h) where cx is the input, cy is the output and w is the weight and h is the score. The scores contain confidence values for 20 objects, and the background value is reserved as 0. Here, the extracted features are used as an arbitrary backbone and the resolution is calculated by reducing the extra layers. Then, this model computes the resolution by concatenating six levels of output. Finally, by utilizing non-maximum suppression (nms), the bounding boxes are filtered out. For a better understanding of the interface, users should first read the manual instruction (guide) of how the YogGuru-Ai works and how efficiently the pose can be performed. The user first needs to select the pose according to their benefit and need. The user should then face the camera. For proper functioning and to obtain good accuracy of the model, the user should practice yoga in a good lighting environment. Figure 3 depicts the Mobile-Net architecture details.

**Figure 4:**
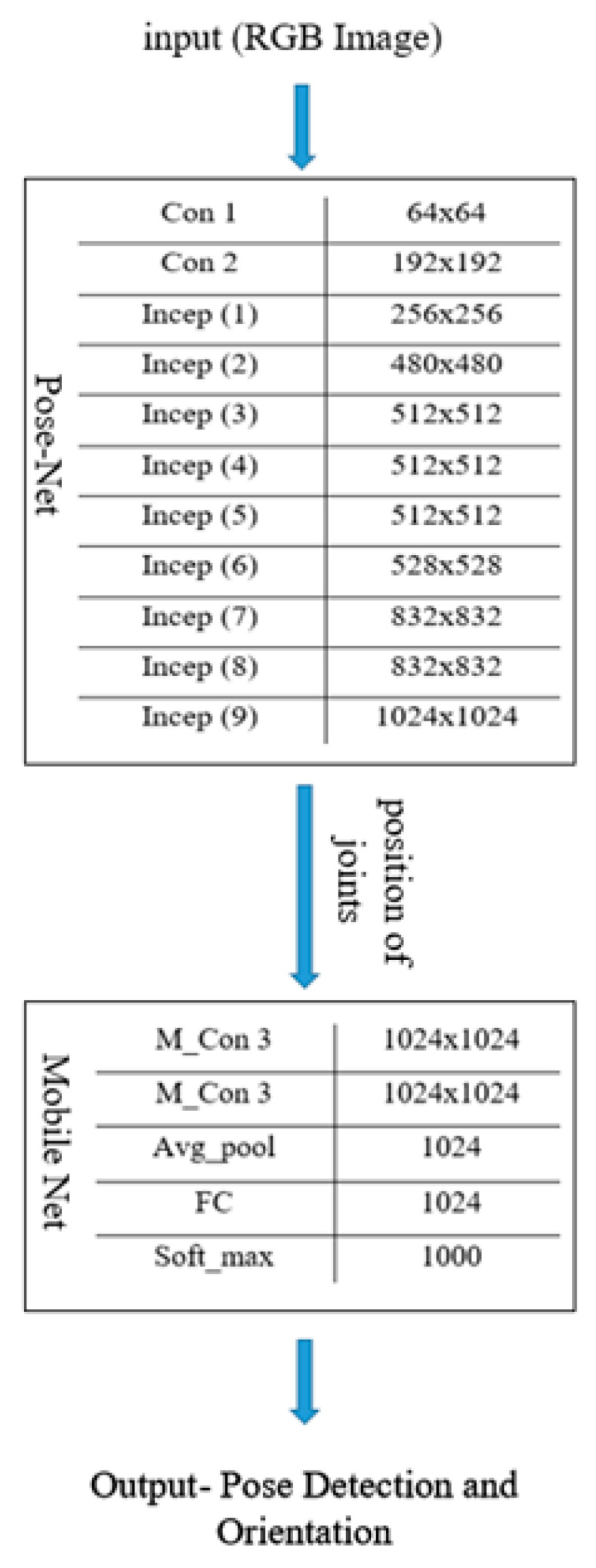
Block level details of Pose-Net and Mobile-Net and their respective output.

After the correct imitation of the pose, the counter begins. The sound of the counter timer motivates the yogi or the user to perform yoga for a longer period of time. If any difficulty is faced by the user while performing the pose, the user can visit the guidelines section for seeing the solution through the FAQ. Table 1 shows the various layers involved in the Y_PN-MSSD implementation. The outcome of the Pose-Net is to locate the position of various joints, while the role of Mobile-Net will be to determine the specified pose and validate the orientation and pose by connecting the joints. Figure 4 shows the role and block specific output of Pose-Net and Mobile-Net networks in determining the right yoga posture.

**Figure 5:**
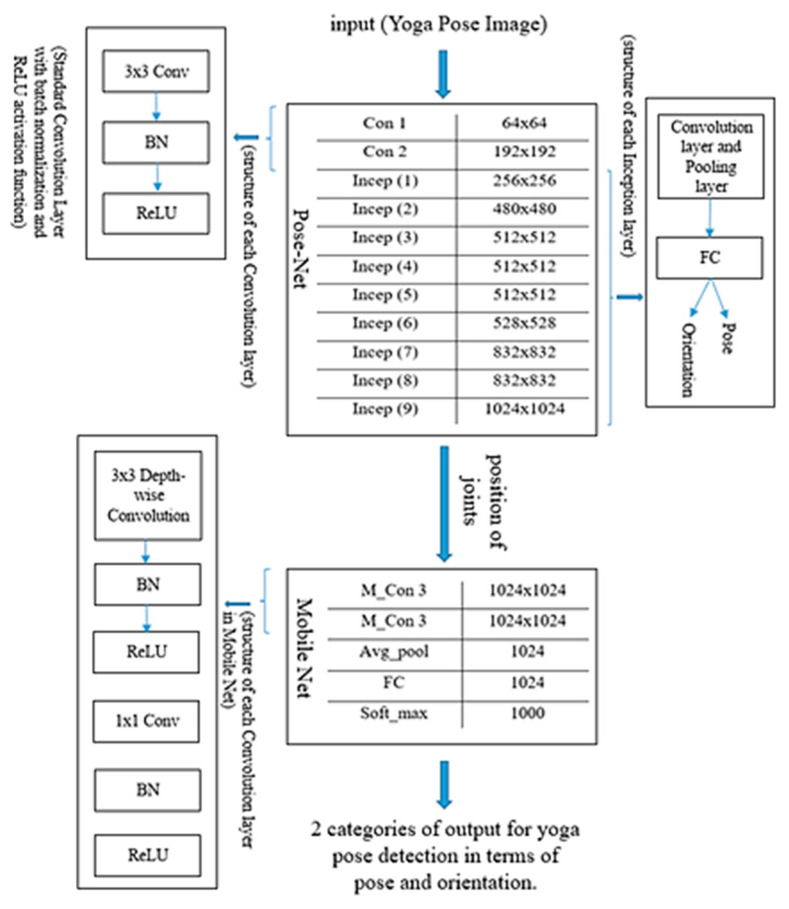
Block diagrammatic representation of the architectural flow of the proposed model.

The input image is fed into the convolution layer (Con), where the edges are enhanced and embossed. Then, the output of convolution layer is fed to the Inception Layer (Incep), where a combination of layers (namely, 1 × 1 Convolutional layer, 3 × 3 Convolutional layer, 5 × 5 Convolutional layer) with their output filter banks concatenate the input into a single output vector (position of joints), which acts as an input of the next layer. The convolution layer of Mobile-Net (M_Con3) handles the output vector for depth-wise convolution. The separated output vectors are further fed into the average pooling layer (Avg_pool), which reduces the dimensions. In the fully connected layer, the output of the previous layer is multiplied with a weighted matrix and a bias vector is added. Finally, the soft max layer (soft_max) converts the vector scores based on the normalized probability distribution to detect the pose of the subject along with its orientation. Figure 5 shows the detailed structure of each layer in the proposed model for determining the right yoga posture.

**Figure 6:**
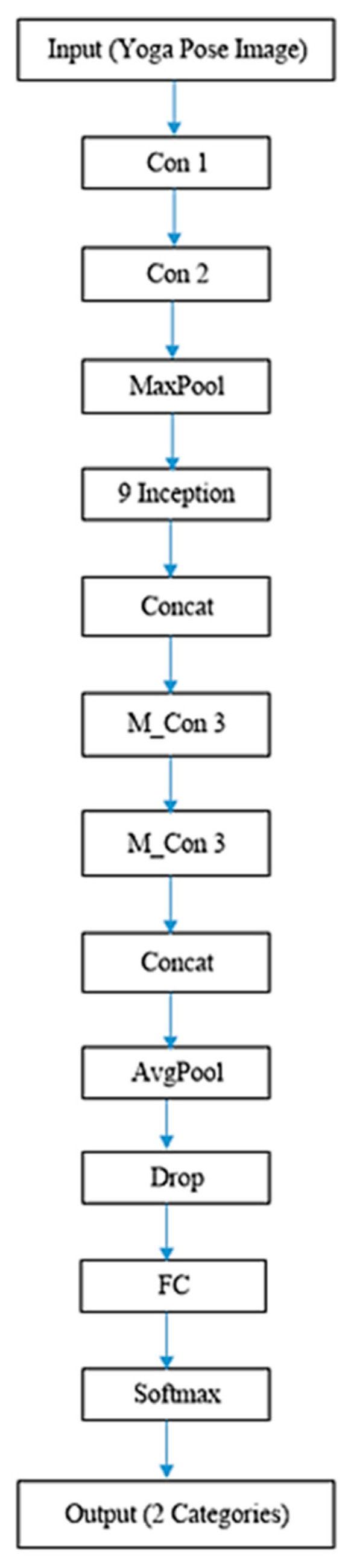
Block representation of the proposed Y_PN-MSSD model.

**Figure 7:**
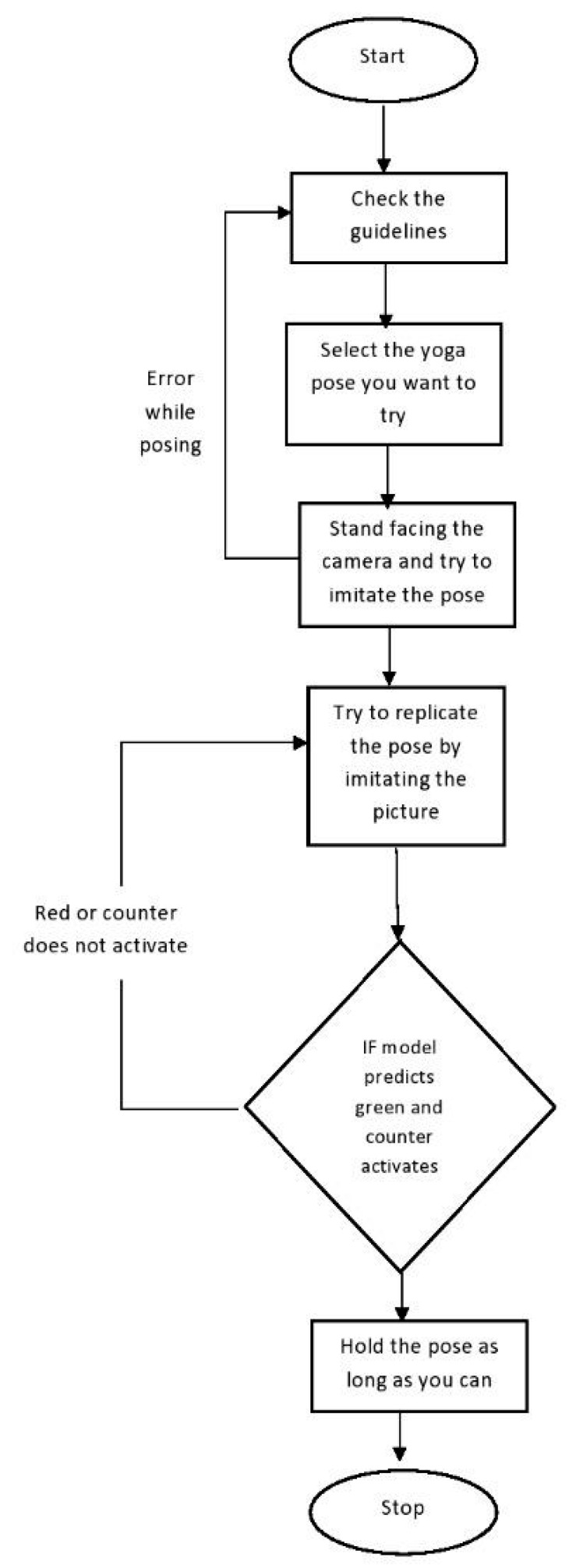
Working of Y_PN-MSSD model with respect to the user interface.

The flow diagram pertaining to the working of this proposed Y_PN-MSSD approach has been depicted in Figure 6 and in terms of user interface has been depicted in Figure 7.

### 3.3. Dataset

**Figure 8:**
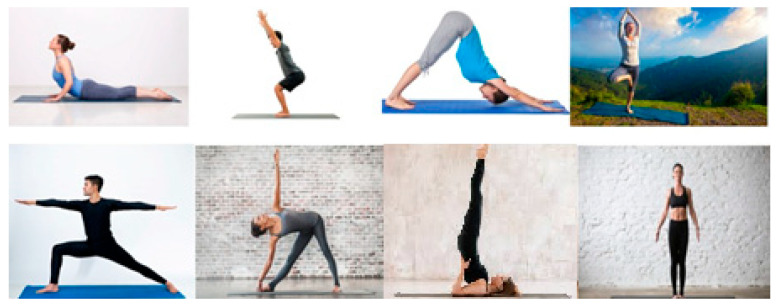
Various frames of yoga postures (cobra, chair, dog, tree, warrior, triangle, shoulder stand, neutral) in dataset used for training the Y_PN-MSSD model.

The proposed model has been trained and tested on an open-source dataset [23] that is comprised of seven yoga poses: Cobra, Chair, Dog, Shoulder stand, Triangle, Tree, and Warrior. This dataset can be accessed using the following link: https://www.kaggle.com/datasets/amanupadhyay/yoga-poses (accessed on 2 January 2023). There are 70 total films of the seven positions, and 350 total instances of the seven poses. About 150 photographs were scraped for each posture from the internet and added to our own datasets. These films were recorded with a camera at a distance of four meters in a room with a frame rate of thirty frames per second. The frame rate can also be increased to a higher value and this will not have any significant bearing on the outcome. Four individuals performed these positions with little modifications in order to obtain robust trained models. Figure 8 shows an example of each pose. It must also be noted that no-pose data images are also taken into consideration. Here, the first image belongs to a no-pose dataset category and later the images of tree, shoulder stand and triangle are in the first row, whereas the image data of chair, cobra, dog and warrior postures are in the later row. Though some of the images in the dataset are in different orientations, they are adjusted by keeping the yoga mat as the reference and subsequent adjustments are made before subjecting the images for training. Images without regular orientation and not having a yoga mat are not considered for training and testing. When the yoga mat is not recognized, an automated alert prompting the user to practice yoga using a mat is generated by the model as shown in Figure 8. This way, the orientation of images is nullified. For both training and testing, the dataset has been used with 320 instances and 30 instances. For testing purpose, various films at different time intervals of data are built and called a secondary dataset. There are 30 examples in this unique dataset with 50 images for each posture.

### 3.4. Real-Time Pose Estimation

In computer vision [24], human pose estimation is a major topic that has had significant progress in recent years from 2D-based single-person pose to multiple-person pose estimation. In general, algorithms [25] estimate poses by detecting key points of the body on an image or video and connecting them to generate output. Here, the x and y coordinates are considered as key points. Keras has been used for real-time multi-person pose estimation to extract critical points to estimate different postures. Each video is subjected to posture estimation. Each pose has been calculated for five consecutive frames, and each frame is taken every two seconds per video. It generates an array of 18 key points [26] for each pose with x and y coordinates. The pose estimation function extracts essential points from a frame, as shown in Figure 8.

**Figure 9:**
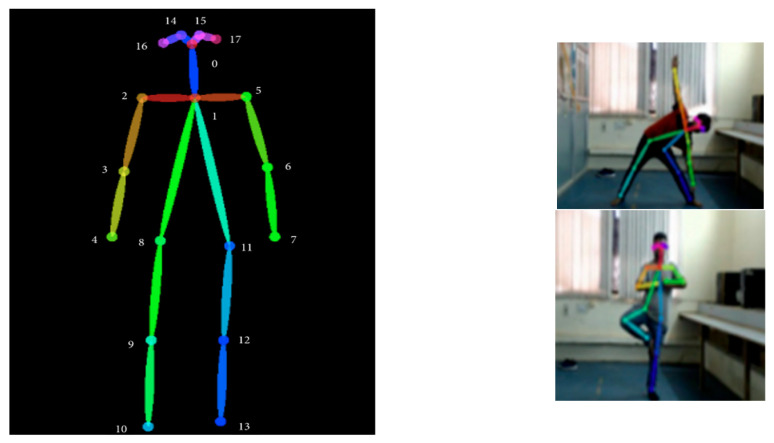
Feature extraction using Y_PN-MSSD model.

The code generates a two-dimensional array in the form of a dictionary, which has keys that represent the coordinates of body parts and values. If several values are identified for a key in the dictionary, then all information has to be displayed, along with their respective levels of confidence. Even if the confidence level is low in comparison to other values, the first identified body point present in the dictionary is considered. As a result, the code must be updated based on a high level of confidence by choosing its value (example: in the tree or triangle pose) as depicted in Figure 9. Here, by using different confidence levels, six body points are detected, namely two arms, two leg joints, i.e., upper thighs, and two being the ends of the legs or the feet.

Many crucial points are observed with varying levels of confidence while detecting poses for a person. Keras pose estimation works by including the first key point found without taking confidence intervals into account. A few tweaks to the Keras posture estimation [27] were made in this work to account for important places with the highest confidence levels. The study used these x and y coordinates to extract characteristics such as angles between body joints and the ground, allowing models to be trained to reach high accuracy. These cases are given top consideration in order to ensure that no anomalous data are used as input. In this work, we have used cross-entropy as the cost function to gauge how well the model performs for the given dataset comprised of seven yoga poses. Cross-entropy can be defined as a measure of the difference between two probability distributions for a given random variable or set of events. The cross-entropy function between two probability distributions A and B can be stated formally as:

H(A, B) = −sum x in X P(x) × log(Q(x))(1)

where P(x) is the probability of the event x in P, and Q(x) is the probability of event x in Q. A margin of error typically lets us know by how many percentage points the actual results obtained differ from the ideal value. In this case, the correct yogic pose is provided by the animated image and it is matched with the live pose frame. The joint positions are identified and lines are drawn. When comparing the joint positions of the live frame and animated image, a deviation of up to 5% is permitted. The Algorithm 1 for pose estimation is shown below:
**Algorithm 1:** Estimating correct pose***Input:****Video, which is converted into frames and fed as images.****Step 1:****Feed the input video. Convert it into N frames.****Step 2:****Use the real-time multi-person pose estimation in Keras.****Step 3:****Extract critical points to estimate posture.****Step 4:****Set frame rate as 2 fps and estimate pose for five consecutive frames.****Step 5:****Generate an array of 18 key points (p1, p2, …., p18) for each pose with x and y coordinates.****Step 6:****Form a dictionary D with these key points. {D} = {p1, p2, p3, ….., p18}****Step 7:****Update the confidence values for the key points.****Step 8:****Use these key points to detect arms, knees, joints, etc.****Step 9:****Test the video frame with the model trained using these key points to estimate the correct posture.**Let {Dv} be the dictionary generated by the input video. Let {Da} be the dictionary generated for a particular yoga pose (Yi) specified by the animated image (Ia).**Compare {Dv} and {Da}.*○*If {Dv} = {Da} => Yi (Yoga pose recognized) => All connected lines become green.*○*If {Dv} ≠ {Da} => Yi not matched => The connected lines remain white.**The user adjusts their pose according to the image until their pose matches with the yoga pose (Yi) specified by the animated image (Ia). [till {Dv} = {Da} situation is satisfied]****Output:****Predicting whether the right pose is attained.****Step 10:****Stop execution.*

### 3.5. Interface

**Figure 10:**
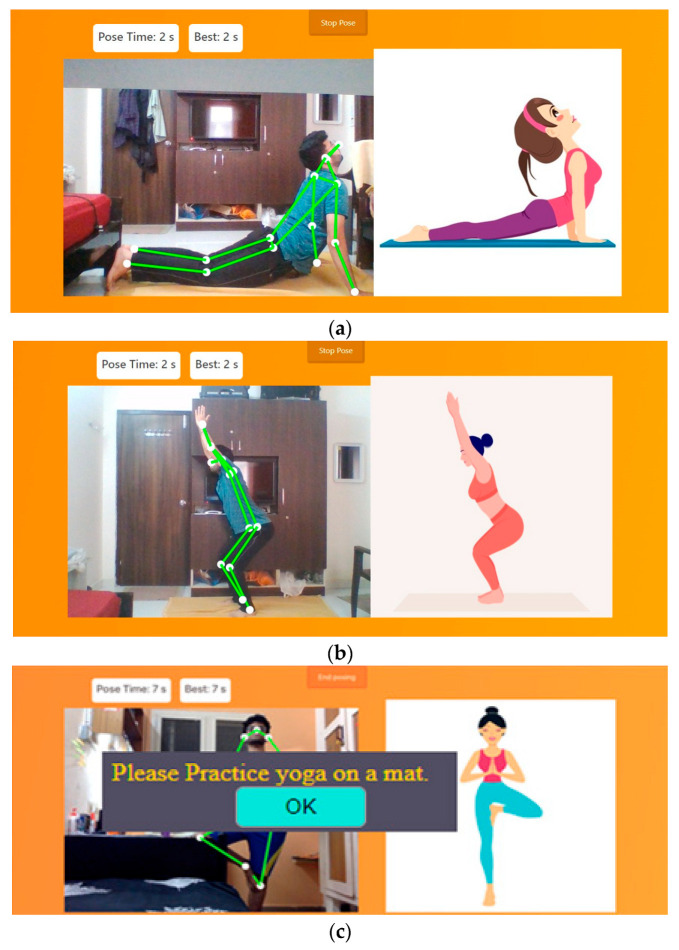
(**a**) Cobra pose recognition by Y_PN-MSSD model, (**b**) chair pose recognition by Y_PN-MSSD model, and (**c**) alert generated when a yoga mat is not recognized.

The interface was designed [28] with a view to help users see whether the yoga pose performed by them is correct or not. Therefore, a menu to select the yoga pose that the user would like to perform is provided. On selecting the required pose, the model will open an inbuilt webcam to analyze the pose. Here, the model Pose-Net in combination with Mobile-Net SSD are loaded in the background with much training performed on it. This recognizes the pose that the user performs through the webcam. In Figure 10, we can see that a tree pose (Figure 10a) has been performed by the user and similarly the chair pose (Figure 10b) has been enacted, and thus the model detects the pose. The model then checks whether the pose that user has enacted is right or not. If it matches with the training data of the required pose, then the connecting line goes green in color, otherwise it will remain in white. It will not turn green unless the user has performed the correct posture. Hence, the model can tell where the user goes wrong and simultaneously a timer is incremented until the pose is performed correctly. However, the timer stops if the user breaks the pose structure. This helps to record how much time the user has stood in the correct yoga pose. This will in turn help the user to improve next time. The user interface also shows the basic guidelines to perform the yoga pose. For performing any pose, this is the sequence of instruction the user should follow. If the user does not practice yoga on a mat, an automated alert is generated, prompting the user to practice yoga on a mat. This is depicted in Figure 9. The basic flow is as follows:

(a) When the app asks for permission to use the camera, allow it to access and capture the pose.
(b) Select what pose the user wants to perform using the dropdown.
(c) Read the instructions of that pose so that the user will know how to perform that pose.
(d) Click on Start pose and see the image of that pose on the right side and replicate that pose in front of the camera.
(e) If the user performs the correct pose, the skeleton over the video will turn green in color and a sound will be played.

**Figure 11:**
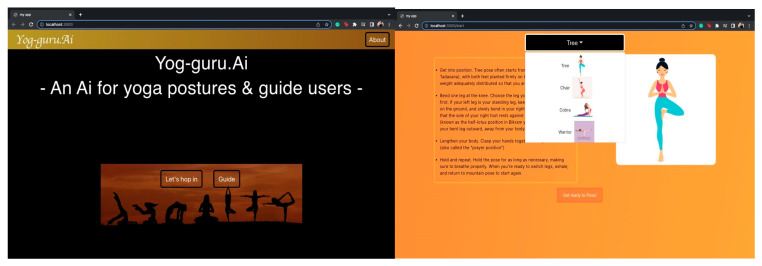
The home page and the user guide on various poses of the Y_PN-MSSD model.

The described model uses Pose-Net and Mobile-Net for human pose prediction. For the ease of the user, the model has an easy-to-use graphical interface. The interface is made into an application with the help of JavaScript. The interface has been developed with ReactJS and an amalgamation of a plethora of json, php, and html scripts. Figure 11 shows the main home page that will be seen by the user. For users’ convenience, the model divides the webpage into categories: 1. Working (“Lets hop in”) and 2. Guide. The application also presents a guided menu wherein the user can select the yoga exercise of their choice. They can also see that each posture name has a guided figure attached within the menu display bar.

**Figure 12:**
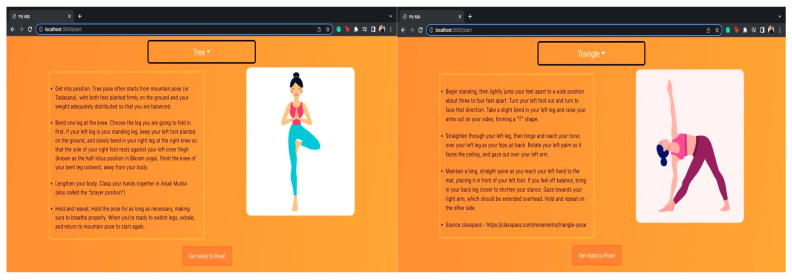
Tree posture and Triangle posture along with a proper guide on how to perform them.

Figure 12 shows tree and triangle yoga postures, respectively. Additionally, some facts and ways to perform certain yoga poses are also described in a side note. This will guide the user on how to carry out a certain pose even if any user is unaware of the yoga asana. In addition, there is a guide page on how to deal with hardware or camera issues on browser. Some of the potential issues faced during the experiment were occlusion and illumination changes. These challenges will be addressed in future work.

## 4. Experimental Results

**Figure 13:**
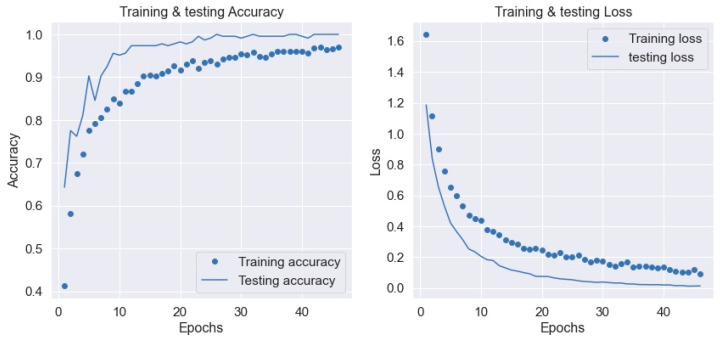
Accuracy and loss of Y_PN-MSSD model when tested in real time.

**Figure 14:**
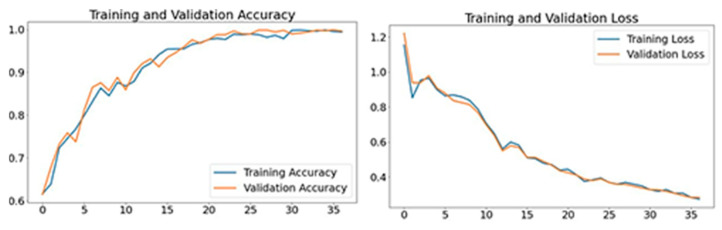
Accuracy and loss of Y_PN-MSSD model when tested with same dataset.

The proposed model provided in this paper uses the layer of a deep learning model to detect wrong yoga postures and correct them to improve. The vectors for nearby joints are used for estimating the angles. The extraction of feature points for pose estimation techniques are characterized in this work. These characteristics are then entered into categorization systems, which give feedback for the correctness of the yoga pose. As a result, the work is split into three parts: (1) feature extraction and time computation for every frame, (2) recognition, and (3) feedback generating time per frame for categorizing yoga poses. Each method’s extraction and computation of features take the same amount of time. This experiment has been performed using NVIDIA GeForce GTX-1080 and Xeon(R) CPU E3-1240 v5. While training and test datasets consist of numerous ups and downs in accuracy until the 200 epoch, an accuracy of 0.9988 was finally attained after 200 epochs. The loss of training and testing dropped gradually after 150 to 200 epochs. This results in coming up with a high confidence training model for classifying yoga postures. The accuracy and loss values have been depicted in Figure 13. It must be noted that the training was carried out using a dataset that contained frames taken from an open-source dataset and also data captured from four users performing seven yoga poses. These two entities constituted the training dataset. The testing was performed using the real time video captured by the user. Hence, the training accuracy is lower than the validation accuracy. The validation accuracy being higher than the training accuracy is a good indicator that the model performs very well in a real-time scenario. Additionally, another approach was carried out to validate the performance of the model in which the dataset was split into training and validation data in the ratio of 80:20. The training and validation sets were mutually exclusive. The accuracy plot for this approach is depicted in Figure 14. It can be seen that the validation accuracy was closely following the training accuracy and there were no major deviations between the curves. Additionally, the loss was decreasing significantly with increasing epochs, which once again confirmed the robust result of the model in terms of accuracy of classification.

**Figure 15:**
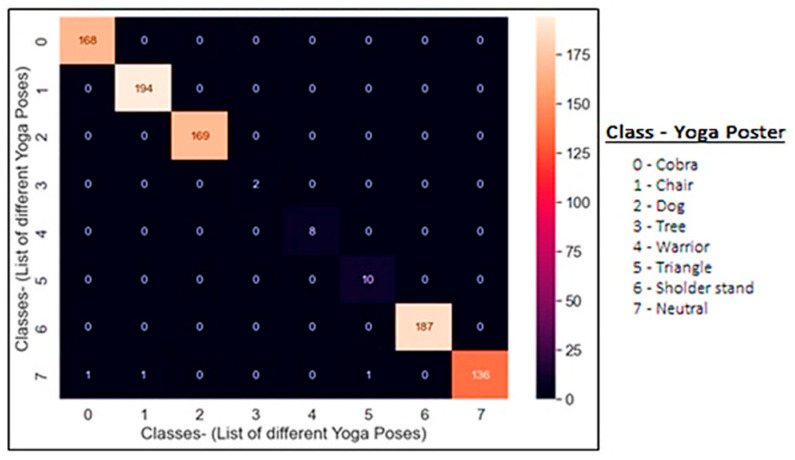
Confusion matrix of Y_PN-MSSD model with predicted labels on x-axis and true labels on y-axis.

The loss of training and test datasets gradually decreased from epoch 0 to 200. The model does not appear to be over fitting based on the training and validation accuracy. Because the research is categorizing input features into one of seven labels, the loss function employed is categorical and the confusion matrix is depicted in Figure 15. This explains the obtained accuracy in a pictorial way, where in the last class-7, the accuracy is slightly fluctuating due to which the obtained accuracy is coming out to be 0.99885976. Table 2 depicts the details of hyperparameters used in the proposed model. An ablation study was carried out to choose the best possible optimizer. For obtaining the desired accuracy, hypermetric tuning was performed in which we have to change the optimizer, namely Adam, AdaDelta, RMSProp, and Adagrad, along with activation function softmax. The comparison of optimizers along with loss and accuracy in 200 epochs is shown in below Table 3. From the table, it can be concluded that the proposed Y_PN-MSSD model with Adam optimizer outperforms the other optimizers.

A confusion matrix is used to evaluate the performance of the proposed yoga recognition model. The frame-based metrics and even score are computed using the following metrics. They are True Positive Rate, False Positive Rate, precision, and recall, respectively. The overall performance is compared with the accuracy of the model. The mathematical model for the given metrics are shown below.

False Positive Rate (FPR) = FP/TN + FP (2)

Precision = TP/TP + FP(3)

Recall, True Positive Rate = TP/TP + FN(4)

Accuracy = TP + TN/TP + TN + FP + FN(5)

**Figure 16:**
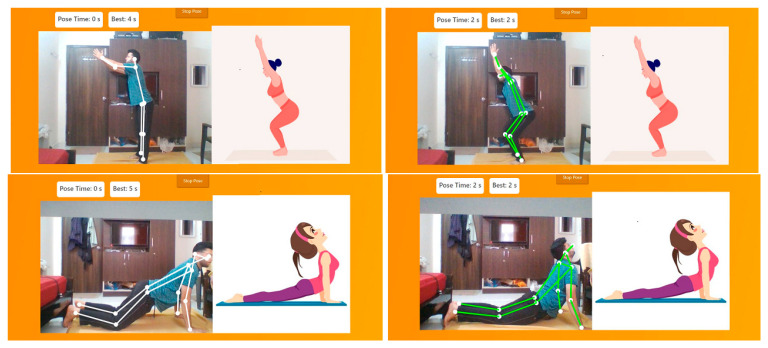
Sample output indicating incorrect pose (white connections) and user on the fly making adjustments until the correct pose (green connections) is attained.

The proposed method is tested with the user pressing the “Get ready to pose” button in the user interface when the video is subsequently captured on the fly. The joints will be located and the connections between joints will be shown in white until the user’s pose matches with that of the pose exhibited by the animated image shown. Once the pose is matched, the connections will turn from white to green, indicating to the user that the correct pose has been attained. The video capture is then turned off by hitting the “stop pose” button in the user interface. To explain this better, some additional sample output images indicating the transition from incorrect pose to correct pose on the fly have been included as Figure 16.

The proposed model result is compared with the existing Pose-Net CNN model with respect to the derived metrics and it is observed that the proposed model outperforms the existing model and is tabulated in Table 4. It is observed that the false positive rate of the proposed model is less when compared to the existing model. Additionally, the precision, recall and accuracy of the proposed model show the best results, and both the models are trained and tested with the same seven yoga posture dataset. Table 5 compares the accuracy of the proposed model with other contemporary works involving yoga posture recognition. It can be seen that the proposed system yields the highest accuracy of 99.88%.

## 5. Conclusions and Future Work

Human posture estimation has been a subject of hot research in recent years. Human posture estimation varies from other computer vision problems in which key points of human body parts are tracked based on a previously defined human body structure. Yoga self-instruction systems have the potential to popularize yoga while also ensuring that it is properly performed. Deep learning models look to be promising because of substantial research in this field. In this paper, the proposed Y_PN-MSSD model is used to recognize seven yoga asanas with an overall accuracy of 99.88%. The accuracy of this model is based on Pose-Net posture assessment and Mobile-Net SSD. A Pose-Net layer takes care of feature point detection, whereas a Mobile-Net SSD layer performs human detection in each frame. This model has been categorized into three stages. In stage one, which is the data collection/preparation stage, the yoga posters are captured from the four users as well as an open-source dataset with seven yoga poses. Then, at the second stage, these collected data have been used for training the model where the feature extraction takes place by connecting key points of the human body. At last, the yoga posture has been recognized. This model will assist the user to perform yoga poses in a live tracking mode and they can correct the posture on the fly. When compared with the Pose-Net CNN model, the proposed model gives better results in terms of accuracy. Additionally, activity recognition is demonstrated in a real-world context and a model such as this could help with pose identification in sports, surveillance, and healthcare in the future. For self-training and real-time forecasting, this model can be used by augmenting the inputs. In future, the yoga posture recognition application can be trained with more number of yoga poses. Additionally, full-fledged training can be performed to build a fully adapted real time model for a real-time noise environment to act as a professional yoga trainer. Future work will be towards addressing other challenges faced during the implementation such as occlusion and illumination changes. Additionally, the proposed model will be enriched to detect more yoga postures. An audio-based alert can be included as part of the future scope to indicate a signal to the user when the correct posture is attained.

## Figures and Tables

**Table 1:** Layer details of the proposed Y_PN-MSSD model.

Layer	Size
Input	224 × 224 × 3 (RGB)
Conv 1	64 × 64
Conv 2	192 × 192
Inception (1)	256 × 256
Inception (2)	480 × 480
Inception (3)	512 × 512
Inception (4)	512 × 512
Inception (5)	512 × 512
Inception (6)	528 × 528
Inception (7)	832 × 832
Inception (8)	832 × 832
Inception (9)	1024 × 1024
M_Con 3	1024 × 1024
M_Con 3	1024 × 1024
Avg_pool	1024
FC	1024
Soft_max	1000
Output	Pose
Output	Orientation

**Table 2:** Hyperparameters of the proposed Y_PN-MSSD model.

Hyperparameters	Values
Image input shape	224 × 224
Channel	3
Batch size	64
Epochs	200
Optimizer	Adam
Loss function	cross-entropy
Learning rate	$10^{-3}$
Minimum learning rate	$10^{-6}$

**Table 3:** Performance of Y_PN-MSSD model based on different optimizers.

Sl. No	Optimizer	Epochs	Loss	Accuracy
1	Adam	200	0.00513306	0.99885976
2	AdaDelta	200	1.95524644	0.45952108
3	RMSProp	200	0.01959268	0.99087798
4	Adagrd	200	1.35576283	0.52793616

**Table 4:** The confusion metrics of the proposed model (Y_PN-MSSD) and existing model (Pose-Net CNN).

Model	Pose Net CNN [13]	Proposed Y_PN-MSSD
FPR	0.03	0.01
Precision	0.97	0.98
Recall, TPR	0.98	0.99
Accuracy	0.98	0.99

**Table 5:** Comparison of accuracy of proposed system and other works involving yoga posture recognition.

Model	Model Used	Best Accuracy Obtained in Each Work
[27]	Distributed CNN, 3D-CNN	96.31%
[26]	TL-VGG16-DA, TL-VGG19-DA, TL-MobileNet-DA, TL-MobileNetV2-DA, TL-InceptionV3-DA, and TL-DenseNet201-DA	98.43%
Proposed System	Y_PN-MSSD	99.88%

## Data Availability

The data used to support the findings of this study are included and will be accessible using the following link https://www.kaggle.com/datasets/amanupadhyay/yoga-poses (accessed on 2 January 2023).
